# Peptide/β-Peptoid Hybrids with Activity against Vancomycin-Resistant Enterococci: Influence of Hydrophobicity and Structural Features on Antibacterial and Hemolytic Properties

**DOI:** 10.3390/ijms22115617

**Published:** 2021-05-25

**Authors:** Martin Vestergaard, Bolette Skive, Ilona Domraceva, Hanne Ingmer, Henrik Franzyk

**Affiliations:** 1 Department of Veterinary and Animal Sciences, Faculty of Health and Medical Sciences, University of Copenhagen, Stigbøjlen 4, DK-1870 Frederiksberg C, Denmark; mave@sund.ku.dk (M.V.); bskive@sund.ku.dk (B.S.); hi@sund.ku.dk (H.I.); 2 Latvian Institute of Organic Synthesis, Aizkraukles 21, 1006 Riga, Latvia; ilona@farm.osi.lv; 3 Center for Peptide-Based Antibiotics, Department of Drug Design and Pharmacology, Faculty of Health and Medical Sciences, University of Copenhagen, DK-2100 Copenhagen, Denmark

**Keywords:** enterococci, vancomycin-resistant *Enterococcus faecium*, VRE, peptidomimetics, antimicrobial agent

## Abstract

Infections with enterococci are challenging to treat due to intrinsic resistance to several antibiotics. Especially vancomycin-resistant *Enterococcus faecium* and *Enterococcus faecalis* are of considerable concern with a limited number of efficacious therapeutics available. From an initial screening of 20 peptidomimetics, 11 stable peptide/β-peptoid hybrids were found to have antibacterial activity against eight *E. faecium* and *E. faecalis* isolates. Microbiological characterization comprised determination of minimal inhibitory concentrations (MICs), probing of synergy with antibiotics in a checkerboard assay, time–kill studies, as well as assessment of membrane integrity. *E. faecium* isolates proved more susceptible than *E. faecalis* isolates, and no differences in susceptibility between the vancomycin-resistant (VRE) and -susceptible *E. faecium* isolates were observed. A test of three peptidomimetics (Ac-[hArg-βNsce]_6_-NH_2_, Ac-[hArg-βNsce-Lys-βNspe]_3_-NH_2_ and Oct-[Lys-βNspe]_6_-NH_2_) in combination with conventional antibiotics (vancomycin, gentamicin, ciprofloxacin, linezolid, rifampicin or azithromycin) revealed no synergy. The same three potent analogues were found to have a bactericidal effect with a membrane-disruptive mode of action. Peptidomimetics Ac-[hArg-βNsce-Lys-βNspe]_3_-NH_2_ and Oct-[Lys-βNspe]_6_-NH_2_ with low MIC values (in the ranges 2–8 µg/mL and 4–16 µg/mL against *E. faecium* and *E. faecalis*, respectively) and displaying weak cytotoxic properties (i.e., <10% hemolysis at a ~100-fold higher concentration than their MICs; IC_50_ values of 73 and 41 µg/mL, respectively, against HepG2 cells) were identified as promising starting points for further optimization studies.

## 1. Introduction

Antibiotics constitute one of the most successful classes of therapeutics in human medicine, and they have significantly reduced the morbidity and mortality caused by bacterial infections during the last century [1]. Nevertheless, our capacity to cure bacterial infections is threatened by the emergence and dissemination of antimicrobial resistance (AMR) [2]. Priority lists for drug-resistant pathogens, for which new therapeutics are most urgently needed, have been defined by the World Health Organization and the American Centers for Disease Control [3,4]. Generally, new therapeutics targeting drug-resistant Gram-negative bacteria are of highest importance; however, new therapeutics against certain Gram-positive pathogens, such as methicillin-resistant *Staphylococcus aureus* (MRSA) and vancomycin-resistant enterococci (VRE), are urgently needed as well [3,4].

Enterococci are usually considered to be commensals of the human gastrointestinal tract; however, upon spreading into the bloodstream, they can cause bacteremia and endocarditis that are associated with high mortality [5,6]. *Enterococcus* species are a common cause of nosocomial infections [7], such as urinary tract, device-associated and surgical-site wound infections, as well as bacteremia [5,6]. The most important enterococcal species associated with disease in humans are *E. faecalis* and *E. faecium* [5,6].

*E. faecium* and *E. faecalis* are intrinsically resistant to several antimicrobials (e.g., aminoglycosides, cephalosporins and trimethoprim-sulfamethoxazole), and they readily acquire resistance to most available antibiotics [8], which limits the treatment options for the associated infections [9]. The greatest concern is the prevalence of vancomycin-resistant enterococci (VRE), which in Europe cause >16,000 infections, and are associated with >1000 attributable deaths per year [10], while >54,000 infections and >5000 attributable deaths per year are reported in the US [4]. Approximately 19% of *E. faecium* isolates in Europe are resistant to vancomycin, which represents a dramatic increase from 8% since 2012 [11]. The development of resistance to newer antibiotics, e.g., linezolid, quinupristin/dalfopristin, daptomycin, and tigecycline, has been reported for enterococci [8], which necessitates the discovery and development of new classes of antibacterial agents against VRE strains.

In the last three decades, antimicrobial peptides (AMPs) have been explored extensively as a potential source of leads for new antibacterial agents as alternatives to small-molecule antibiotics [12]. Nevertheless, most AMPs displaying promising antibacterial activity in vitro appear to have mainly been tested in animal models, allowing for topical administration due to inherent toxicity issues with these typically highly amphipathic peptides, and not least because of their low stability toward enzymatic degradation in vivo [12,13]. However, several classes of peptidomimetics incorporating unnatural amino acids or residues resembling amino acids have been shown to possess improved bioavailability profiles and higher metabolic stability than AMPs [14]. The most investigated types of such peptidomimetics comprise: peptoids [15], β-peptoids [16], β-peptides [17], peptide/β-peptide hybrids [18], lipo-AApeptides (i.e., lipidated oligomers based on *N*-acylated-*N*-aminoethyl amino acids) [19], and peptide/peptoid hybrids [20,21,22].

In a previous comparative study of peptidomimetics comprising multiple backbone designs, but displaying a common alternating cationic/hydrophobic design, peptide/β-peptoid hybrids were found to possess superior antibacterial activity profiles, including high cell selectivity [21]. Besides this, representatives of this compound class have also been proven to exhibit activities against food-related bacterial pathogens [23] and growth-inhibitory effects on *Staphylococcus epidermidis* biofilm [24]. Intriguingly, contrary to most AMPs, these peptidomimetics exhibit increased potency when tested in media containing up to 25% plasma [25].

**Figure 1:**
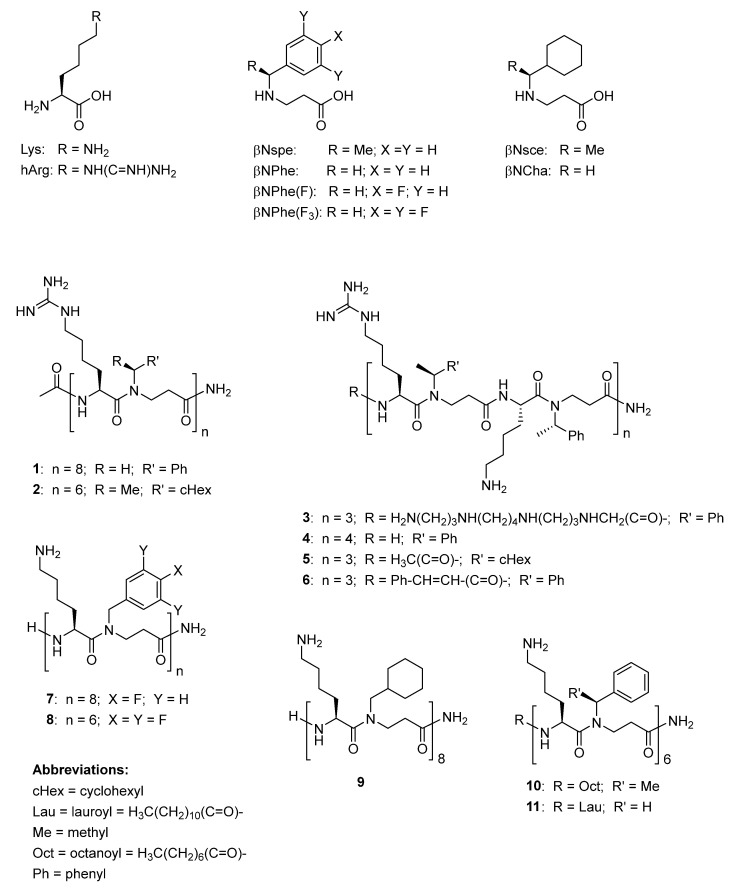
Chemical structures of compounds investigated in the present study.

In the present study, we initially screened 20 peptidomimetics (Table S1), and the 11 most active compounds (Figure 1 and Table 1) were selected for further investigation of their antibacterial activity against eight *E. faecium* and *E. faecalis* strains, including ATCC reference strains and three clinical vancomycin-resistant *E. faecium* isolates of human origin. We further characterized the selected representative compounds in terms of mode of action, hemolysis and effect on mammalian cell viability.

## 2. Results and Discussion

### 2.1. Structural Diversity of Studied Peptidomimetics

The investigated peptidomimetics, all having an alternating cationic/hydrophobic design, can be divided into three subclasses (I, II and III; Table 1), based on the nature of the side-chain functionalities of the cationic residues: (I) guanidino only, displayed by side chains of homoarginine (hArg; i.e., **1** and **2**), (II) guanidino and amino, displayed by lysine (Lys) and hArg in a 1:1 ratio (i.e., **3**–**6**), and (III) amino only, displayed by Lys (i.e., **7**–**11**). The hydrophobic residues are represented by simple β-peptoid analogues of phenylalanine (Phe) with an achiral side chain (i.e., βNPhe in **1** and **11**) or chiral side chains (i.e., βNspe in **3**–**6** and **10**), β-peptoid analogues of fluorinated phenylalanine (i.e., βNPhe(F) in **7** or βNPhe(F_3_) in **8**), or by β-peptoid analogues of cyclohexylalanine (Cha) with achiral or chiral side chains (i.e., βNCha in **9** or βNsce in **2** and **5**). Previously, some of these peptidomimetics were included in screenings against ESKAPE pathogens (*Enterococcus faecium*, *Staphylococcus aureus*, *Klebsiella pneumoniae*, *Acinetobacter baumannii*, *Pseudomonas aeruginosa* and *Enterobacter* spp. [26]), and **1**–**6** were found to have minimal inhibitory concentrations (MICs) in the range 1–8 µg/mL against the VRE strain *E. faecium* ATCC 700221 [20,27], while **7**–**9** were found to have MIC values within the range 2–4 µg/mL toward the VRE strain *E. faecium* BM4147 [28]. In contrast, the lipidated analogues **10** and **11**, displaying an *N*-terminal fatty acid, have only been investigated with respect to their immunomodulatory activity [29,30].

**Table 1:** Properties of peptidomimetics (oligomer length, molecular weight and net charge).

No.	Peptidomimetic	Length (Residues)	*M*_W_ ^1^ (g/mol)	Charge	Ref.
Subclass I					
**1**	Ac-[hArg-βNPhe]_8_-NH_2_	16	3622.56	+8	[20]
**2**	Ac-[hArg-βNsce]_6_-NH_2_	12	2852.13	+6	[20]
Subclass II					
**3**	SpermineAc-[hArg-βNspe-Lys-βNspe]_3_-NH_2_	13	3346.14	+10	[27]
**4**	H-[hArg-βNspe-Lys-βNspe]_4_-NH_2_	16	3638.59	+9	[27]
**5**	Ac-[hArg-βNsce-Lys-βNspe]_3_-NH_2_	12	2707.87	+6	[20]
**6**	Cinn-[hArg-βNspe-Lys-βNspe]_3_-NH_2_	13	2777.83	+6	[27]
Subclass III					
**7**	H-[Lys-βNPhe(F)]_8_-NH_2_	16	3502.20	+9	[28]
**8**	H-[Lys-βNPhe(F_3_)]_6_-NH_2_	12	2875.29	+7	[28]
**9**	H-[Lys-βNCha]_8_-NH_2_	16	2671.92	+9	[28]
**10**	Oct-[Lys-βNspe]_6_-NH_2_	13	2647.76	+6	[29]
**11**	Lau-[Lys-βNPhe]_6_-NH_2_	13	2619.71	+6	[30]

^1^ Molecular weight including TFA counter ions. Abbreviations: Ac = acetyl; Cinn = cinnamoyl; hArg = homoarginine; Lau = lauroyl; βNCha = *N*-cyclohexylmethyl-β-alanine; βNPhe = *N*-benzyl-β-alanine; βNPhe(F) = *N*-(4-fluorobenzyl)-β-alanine; βNPhe(F_3_) = *N*-(3,4,5-trifluorobenzyl)-β-alanine; βNsce = *N*-(*S*)-1-cyclohexylethyl-β-alanine; βNspe = *N*-(*S*)-1-phenylethyl-β-alanine; Oct = octanoyl; SpermineAc = H_2_N(CH_2_)_3_NH(CH_2_)_4_NH(CH_2_)_3_NHCH_2_(C=O)-.

### 2.2. Peptidomimetics Inhibit Growth of Enterococci, Including Vancomycin-Resistant E. faecium

Initially, a wider compound collection comprising 20 peptidomimetics was screened for growth-inhibitory activity against multiple enterococcal isolates from both human and veterinary sources at a fixed concentration of 32 µg/mL, with incubation for 24 h and 48 h (Table S1). The 11 most active peptidomimetics were selected for determination of their MICs against five isolates of *E. faecium* (including three clinical vancomycin-resistant isolates of human origin) and three vancomycin-sensitive *E. faecalis* isolates.

The minimum inhibitory concentrations for the 11 peptidomimetics ranged from 2 to >32 µg/mL (Table 2). Generally, *E. faecium* was more susceptible to the peptidomimetics than *E. faecalis*. Importantly, there were no differences in susceptibility to the peptidomimetics between the vancomycin-resistant and -susceptible *E. faecium* isolates.

While the hydrophobicity of small molecules is typically estimated by their partition coefficients, e.g., the log D values, the relative retention in reverse-phase HPLC (RP-HPLC) constitutes a more appropriate measure of hydrophobicity for very polar and highly cationic peptides and peptidomimetics [15,18,31,32]. Recently, within a large set of peptidomimetics, resembling those in the present study, we identified a correlation between hydrophobicity, expressed as percent acetonitrile (%MeCN) at peak elution in analytical RP-HPLC, antibacterial activity, and hemolytic properties [32]. Here, it proved possible to identify a hydrophobicity threshold above which cell selectivity was partially lost (i.e., >10% hemolysis at 400 µg/mL) for 12-mer α-peptoid/peptide hybrids displaying α-peptoid lysine-like cationic residues and hydrophobic α-amino acids. For that subclass, this threshold (43.9% MeCN) coincided with the hydrophobicity threshold required to confer potent activity against *E. faecalis*. In contrast, for Gram-negative bacteria, the latter lower hydrophobicity thresholds for antibacterial activity were considerably lower, implying that an appropriate design window for compounds with intermediate hydrophobicity existed [32]. These previous results indicated that it might be quite difficult to identify compounds displaying anti-enterococcal activity while possessing satisfactory cell selectivity.

Intriguingly, a similar analysis of the hydrophobicity of the present 11 peptidomimetics (all having an alternative peptide/β-peptoid design with cationic amino acids) revealed that they cover a wide range of hydrophobicity (from 40.2% to 51.6% MeCN (Table 3) when using the same setup as for analytical HPLC [32]). Moreover, several compounds display acceptable hemolytic properties (i.e., less than 10% hemolysis at 400 µg/mL, corresponding to a ~100-fold higher concentration than the typical MIC values against *E. faecium*), while other analogues possess considerable hemolytic activity (Table 3). In the following, the influence of hydrophobicity (and the structural features related to this) on both antibacterial and hemolytic properties will be discussed for each subclass and across the entire set of compounds.

Overall, the most active compound (MICs of 2–4 µg/mL) against all *E. faecium* and *E. faecalis* isolates was analogue **2**, belonging to subclass I. This compound displays both the strong hydrogen-bonding guanidino groups and the highly hydrophobic aliphatic cyclohexyl side chains (in βNsce), conferring a peak elution at 51.6% MeCN in RP-HPLC, corresponding to the highest hydrophobicity within the entire set. Hence, **2** possesses a molecular design with a very high propensity to exert unspecific membrane-disruptive activity, as also reflected in its pronounced hemolytic properties giving rise to almost complete lysis of erythrocytes at 400 µg/mL. While the other guanidino-containing analogue (i.e., **1**) retained almost full activity (MICs of 2–8 µg/mL) against *E. faecium,* it had significantly reduced potency toward *E. faecalis* with MICs in the range 16 to >32 µg/mL. This appears to be a result of replacing the βNsce residues with the less hydrophobic aromatic βNPhe units, conferring a significantly lower overall hydrophobicity (~peak elution at 41.8% MeCN). Expectedly, compound **1** exhibited relatively low hemolysis (i.e., ~6% at 400 µg/mL) as a result of its relatively low hydrophobicity.

In subclass II, analogue **5** is the only member that is almost equipotent (with a typical MIC of 2 µg/mL) to compound **2** against *E. faecium*. In contrast, analogues **3** (with increased positive charge), **4** (with a longer oligomer length) and **6** (displaying an *N*-terminal hydrophobic cinnamoyl moiety) all possess slightly reduced potency (typical MICs in the range 4–8 µg/mL) against *E. faecium*. Moreover, compound **6** has an activity profile (all MICs 16 µg/mL) similar to that of analogue **5** (MIC range of 4–16 µg/mL) against *E. faecalis*, whereas analogues **3** and **4** exhibit lower activity (MICs in the range 16 to >32 µg/mL) toward this species.

A high content of hydrophobicity-inducing residues/modifications is required for efficient interaction with the thick bacterial cell wall of Gram-positive species. Thus, **5** and **6** contain three βNsce residues and an *N*-terminal modification with cinnamic acid, respectively, both conferring the hydrophobicity needed (i.e., elution at 45–47% MeCN). However, increased antibacterial activity induced by a higher hydrophobicity usually comes at a cost of concomitantly increased hemolytic properties, as seen when comparing analogues **3**–**6**, for which increasing the hydrophobicity from 40% (for **3**) to ca. 46–47% MeCN (for **5** and **6**) induced a pronounced rise in hemolysis from 0.6% to 15.7% at 400 µg/mL. This finding is in accordance with a recent report on a wide range of peptoid/peptide hybrids [32].

In subclass III, only amino groups contribute to the cationicity, while hydrophobicity arises from either fluorinated aromatic side chains (i.e., in **7** and **8**), βNCha residues (in **9**), or *N*-terminal lipidation with fatty acids (in **10** and **11**). Most members (i.e., **8–11**) of this subclass exhibited potent activity (MICs typically in the range 2–4 µg/mL) against *E. faecium*, while only **9** and **11** retained similar potency toward *E. faecalis* isolates. Compound **7** was the least hemolytic in this subclass, but displayed only medium to low antibacterial potency (i.e., MICs of 8 to >32 µg/mL) against the *E. faecium* ATCC 19434 strain and all *E. faecalis* isolates, despite being one of two longer oligomers (i.e., **7** and **9**) in this group. Both **8** and **9,** as well as **11** in particular, possessed undesirably pronounced hemolysis at 400 µg/mL (i.e., above 20%)—most likely arising from their high content of hydrophobicity-inducing moieties (conferring a peak elution above 45% MeCN). Overall, compound **10** possessed the best balance between antibacterial potency and hemolytic properties in this subclass, although compound **7** performed quite similarly, except for its high MIC (>32 µg/mL) toward the *E. faecalis* 38262 isolate. Thus, a limited degree of fluorination (in **7**) and *N*-terminal modification with a short fatty acid (in **10**) both confer sufficient hydrophobicity for antibacterial activity without enhancing the hemolysis to an unacceptable level.

Across all three subclasses, analogue **2** was found to be the most potent antibacterial compound, albeit being very toxic to erythrocytes, while analogues **5** and **10** possessed the most promising balance between antibacterial activity and hemolytic properties.

In addition, all compounds were examined with respect to their effect on the viability of HepG2 cells (Table 3). Somewhat surprisingly, **5** proved to affect viability to the lowest degree, as indicated by an IC_50_ value of 73 µg/mL versus 40–50 µg/mL for **3, 4**, **6**, **7** and **10** (all except **6** being less hemolytic than **5**). Thus, hemolytic properties and effect on HepG2 cells appear not to be closely correlated, and therefore arise from different structural features. Noticeably, the compounds exerting the most pronounced detrimental effects on HepG2 cells comprise both subclass I members (i.e., **1** and **2**), displaying the guanidino-containing hArg as the cationic residues, as well as the amino-containing subclass III members **9** (displaying the hydrophobic βNCha residue in a 16-mer) and **11** (displaying a C_12_ fatty acid). Interestingly, the hydrophobicity seems not to be the major determinant for the detrimental effect on HepG2 cells, e.g., **1** and **2** had very different retentions in RP-HPLC (peak elution at 41.8% and 51.6% MeCN, respectively), while both were among the most cytotoxic compounds, most likely reflecting the capability of guanidino groups to form efficient bidentate hydrogen bonding with phospholipids.

The safety window for analogue **5** appears satisfactory for an unoptimized hit compound, as estimated by its selectivity index (SI), calculated as the ratio between IC_50_ (for effect on HepG2 viability) and typical MIC value against *E. faecium* isolates. Thus, the SI for peptidomimetic **5** is approximately 35, indicating a preferential killing of bacteria over human cells even at concentrations well above the MIC. With respect to antibacterial potency, hydrophobicities above ~41.5% and ~45–46% MeCN appear to be required for *E. faecium* and *E. faecalis*, respectively. The cell envelope varies between *E. faecium* and *E. faecalis* in terms of the subunit composition of wall teichoic acids (WTA) [33,34]; however, it remains to be explored whether these differences in WTA composition contribute to the general lower inhibitory activity against *E. faecalis* in comparison to *E. faecium* observed for the peptidomimetics.

### 2.3. No Synergy between Peptidomimetics and Conventional Antibiotics

In a previous study, combinations of peptidomimetics with conventional antibiotics (rifampicin and azithromycin) were found to act synergistically against Gram-negative bacteria, including *E. coli*, *P. aeruginosa* and *K. pneumoniae* [35]. Hence, we also assessed the potential for synergy between three peptidomimetics, comprising the most active compound (i.e., **2**) and the two compounds with the most favorable overall activity profiles (i.e., **5** and **10**), and different classes of conventional antibiotics against enterococci. Since vancomycin-resistant enterococci (VRE) constitute an increasing problem in hospital environments [36], we included an assessment of whether sub-inhibitory concentrations of **2**, **5** and **10** would resensitize two vancomycin-resistant *E. faecium* isolates toward vancomycin; however, this was not the case (Table S2). Likewise, the vancomycin–peptidomimetic combinations proved indifferent against vancomycin-susceptible enterococci.

Enterococci have intrinsic low-level resistance to aminoglycosides due to limited drug uptake, which is attributed to their facultative anaerobic metabolism [37]. Sub-inhibitory concentrations of **2**, **5** and **10** did not sensitize the enterococcal isolates to gentamicin. Similarly, there were no synergistic effects when the peptidomimetics were combined with any of the other tested conventional antibiotics (ciprofloxacin, linezolid, rifampicin or azithromycin) (Table S2). However, when compound **10** was supplemented at 0.5× MIC, a 4–8-fold reduction in the MIC of rifimpicin toward *E. faecium* ATCC 19434 was observed (but with an FICI > 0.5 this does correspond to synergy), while no effect was observed when **10** was supplemented at 0.25× MIC.

These data imply that this class of peptidomimetics does not facilitate the uptake of common conventional antibiotics in enterococci, contrasting a previous observation for rifampicin and azithromycin in Gram-negative bacteria [35].

### 2.4. Compounds ***2***, ***5*** and ***10*** Kill Enterococci

**Figure 2:**
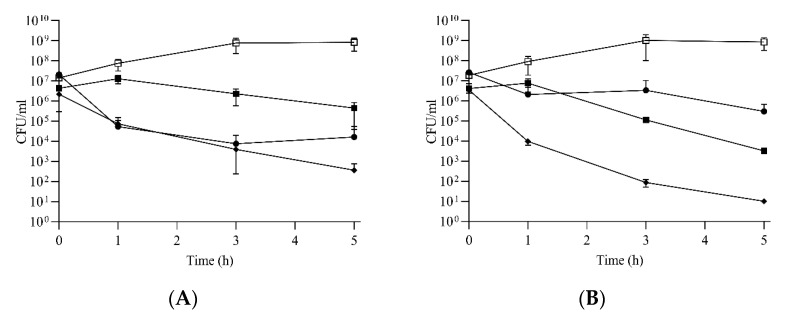
Compounds **2**, **5** and **10** kill enterococci. Time–kill curves for *E. faecium* ATCC19434 (**A**) and *E. faecalis* ATCC29212 (**B**) treated with compound **2** (●), **5** (■) and **10** (♦) at concentrations corresponding to 4× MIC. Growth controls in untreated BHI broth for both strains are also displayed (□). Each time point represents the average of at least three biological replicates and the error bars represent the standard deviation.

For the most potent (but cytotoxic) peptidomimetic **2**, and the somewhat less potent (but less cytotoxic) **5** and **10**, time–kill kinetics against *E. faecium* and *E. faecalis* were assessed to determine whether the compounds act via a bactericidal or bacteriostatic mode of action toward enterococci. Compound **2** proved bactericidal with an approximately 3-log reduction at 8 µg/mL (i.e., 4× MIC) against *E. faecium* (Figure 2A). The killing rate for **2** against *E. faecalis* was lower at 8 µg/mL (i.e., 2–4× MIC), showing an approximately 2-log reduction in viable cells within 5 h. Interestingly, compound **5** showed no reduction in viable cell counts within 1 h of exposure at concentrations corresponding to 4× MIC. Reductions in viable cell counts occurred only after 1 h, and upon 5 h of continuous exposure to **5,** 1.5-log and 3-log reductions in viable bacteria were found for *E. faecium* (Figure 2A) and *E. faecalis* (Figure 2B), respectively. Compound **10** displayed the highest killing rate with 4-log and 5-log reductions in viable bacteria after 5 h against these isolates (Figure 2A,B).

### 2.5. Compounds ***2***, ***5*** and ***10*** Compromise Membrane Integrity to a Different Degree

Peptidomimetics belonging to the subclass of peptide/α-peptoid hybrids have previously been found to act by disruption of membrane integrity in *E. coli* [38], which is similar to the mode of action of most AMPs [12].

**Figure 3:**
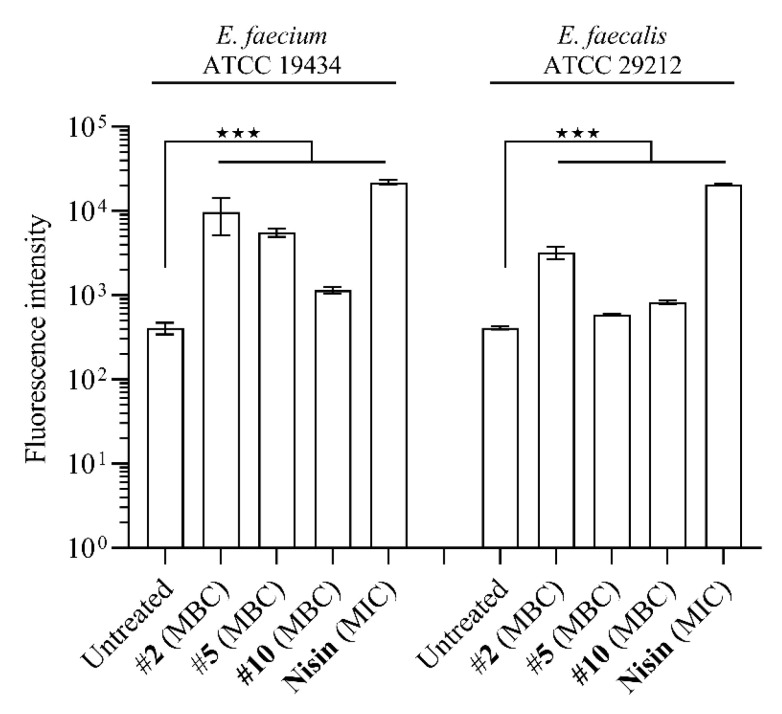
Compounds **2**, **5** and **10** interfere with membrane integrity to a different degree. Membrane integrity was assayed (at MBC; Table S3) by using the fluorescent dye propidium iodide (PI) that only crosses compromised membranes. Each group displays the average of three measurements and the error bars display the standard deviation. ★★★ *p* < 0.001.

Therefore, we assessed whether compounds **2**, **5** and **10** also interfere with membrane integrity in enterococci by measuring propidium iodide (PI) uptake upon exposure to the peptidomimetics at concentrations corresponding to their MBCs (Table S3). Propidium iodide only crosses compromised bacterial membranes [39], and in these experiments the pore-forming AMP nisin was used as the positive control. Exposing *E. faecium* (MV388) and *E. faecalis* (MV269) to compound **2** (at MBC) for 5 min resulted in a significant accumulation of PI, as also observed for cells exposed to nisin (at MIC) (Figure 3). *E. faecium* (MV388) and *E. faecalis* (MV269) cells exposed to **5** and **10** showed less perturbation of membrane integrity as compared to that seen for **2**. Taken together, the data suggest that the modes of action of peptidomimetics **2**, **5** and **10** involve different degrees of membrane perturbation, and likely with substantial differences in the specific interactions.

## 3. Conclusions

Even though the set of 11 peptide/β-peptoid hybrids was structurally diverse and spanned a wide range of hydrophobicity, it appears too limited to allow for the identification of the precise thresholds that confer potent antibacterial and unacceptable hemolytic properties, respectively. Nevertheless, some indicative guidelines for applying hydrophobicity in the selection of novel analogues for microbiological studies can be deduced. Thus, cell selectivity seems to be very limited for analogues with a hydrophobicity above approximately 46–47% MeCN, depending on the nature of the hydrophobicity-inducing features. Combined with the hydrophobicity thresholds (~41.5% and ~45–46% MeCN) for potency against *E. faecium* and *E. faecalis*, respectively, only a narrow design window seems to exist for compounds with general high anti-enterococcal activity without detrimental effects on the viability of host red blood cells. For *E. faecium*, the design options are less restricted by hydrophobicity, as the acceptable range is approximately 41.5–46% MeCN.

Compound **2** was the most potent with MICs in the range 2–4 µg/mL against all isolates, but unfortunately it also proved highly hemolytic. Compound **5** was equipotent to **2** against *E. faecium*, but was less active against *E. faecalis*. Analogues **2**, **5**, and **10** were all found to be bactericidal, which may be a consequence of membrane perturbation, as clearly seen for **2,** but less pronounced for **5** and **10**. Interestingly, compound **10** exhibited the highest killing rate on both the *E. faecium* and *E. faecalis* strains, resulting in the highest reductions in viable bacteria during the time-kill experiments. In addition, analogue **10** exhibited only slightly poorer activity than **5**, but it was marginally less hemolytic, and collectively, compounds **5** and **10** constitute the most promising leads for further optimization studies. 

Structural features, conferring sufficient hydrophobicity for efficient disruptive interactions with enterococcal membranes, which appear most interesting to pursue in future work, comprise the incorporation of a limited degree of fluorination of aromatic hydrophobic residues (e.g., as seen in **7** with an activity profile almost as favourable as **5** and **10**), a certain content of hArg and/or Cha-based peptoid residues (e.g., βNsce as seen in **5**), and *N*-terminal modification with a short fatty acid (present in **10**).

## 4. Materials and Methods

### 4.1. Analytical HPLC

Water was filtered by using an Evoqua LaboStar PRO TWF Ultra Pure Water System prior to use in high-performance liquid chromatography (HPLC). The retention time for each peptidomimetic was determined by analytical HPLC using a Phenomenex Luna C18(2) HST column (100 mm × 3 mm; particle size: 2.5 μm; pore size: 100 Å) on a Shimadzu Prominence and Shimadzu Nexera system using an aqueous MeCN gradient with 0.1% trifluoroacetic acid (TFA) added (eluent A: 5:95 MeCN–H_2_O + 0.1% TFA; eluent B: 95:5 MeCN–H_2_O + 0.1% TFA); a flow rate of 0.5 mL/min was used. For the elution of peptidomimetics, a linear gradient of 0% to 60% B over 10 min was used with UV detection at λ = 220 nm. The percentage MeCN at peak elution was calculated from the retention time (t_R_) by using the following formula:

%MeCN = [0.95 × 0.6 × t_R_/10 + 0.05 × (1 − 0.6 × t_R_/10)] × 100%

### 4.2. Bacterial Strains, Growth Conditions and Chemicals

Five *E. faecium* and three *E. faecalis* isolates were used in the present study (Table S4). Antibiotics (vancomycin, ciprofloxacin, gentamicin, rifampicin, nisin, linezolid and azithromycin) were purchased from Merck KGaA (Darmstadt, Germany), while the collection of peptidomimetics was provided by Henrik Franzyk (Table 1 for characteristics). Stock solutions of the peptidomimetics were prepared by dissolving the compounds in deionized water.

The enterococcal isolates were routinely cultured in cation-adjusted Mueller Hinton broth (MHB II; Merck KGaA, Darmstadt, Germany), in brain–heart infusion broth (BHI; Oxoid, Roskilde, Denmark) or on brain–heart infusion agar (BHA; Oxoid, Roskilde, Denmark). All isolates were cultured at 37 °C.

### 4.3. Determination of Minimum Inhibitory Concentration (MIC) and Minimum Bactericidal Concentration (MBC)

The MICs for the peptidomimetics and conventional antibiotics (vancomycin, ciprofloxacin, gentamicin, rifampicin, linezolid, azithromycin and nisin) were determined by using the two-fold broth microdilution assay. Overnight cultures of the enterococcal isolates were diluted in physiological saline (0.9% NaCl) to reach a turbidity of 0.5 McFarland units (Sensititre^®^ nephelometer and the Sensititre^®^ McFarland Standard). Bacterial suspensions were adjusted to 5 × 10^5^ CFU/mL in MHB II or BHI broth containing two-fold dilutions of peptidomimetics or conventional antibiotics in a final volume of 100 µL. The plates were incubated for 24 h at 37 °C without shaking. The MIC was defined as the concentration of the agent that completely prevented visible growth. All experiments were performed in three biological replicates.

Since BHI better supports the growth of enterococci as compared to MHB II, which is traditionally used for susceptibility studies, we also assessed the MICs of compounds **2**, **5** and **10** against *E. faecium* (MV388) and *E. faecalis* (MV269) when propagated in BHI. The MICs of the three compounds against both isolates were identical in BHI and MHB II. Hence, the following experiments were conducted in BHI medium.

After the MIC determination of compound **2**, **5** and **10**, aliquots of 50 μL from all the tubes that showed no visible bacterial growth were plated on BHI agar plates and incubated for 24 h at 37 °C. The minimum bactericidal concentration (MBC) is defined as the lowest concentration of the peptidomimetics that kills 99.9% of the bacterial population. All experiments were performed in three biological replicates.

### 4.4. Combination with Conventional Antibiotics

Drug interactions between three peptidomimetics (**2**, **5** and **10**) and conventional antibiotics (vancomycin, ciprofloxacin, gentamicin, rifampicin, linezolid and azithromycin) were assessed by using the broth checkerboard assay in BHI broth. The strain culture preparation and final volume were the same as in the MIC assay. The concentrations of the peptidomimetics were 0.25×, 0.5×, 1× and 2× MIC, while the concentrations (2-fold increments) for the conventional antibiotics ranged from 0.125× to 2× MIC. The plates were incubated for 24 h at 37 °C without shaking. MICs were defined as the concentrations of the agents that completely prevented visible growth. All experiments were performed in two biological replicates. 

To evaluate the effect of the combinations, the fractional inhibitory concentration index (FICI) was calculated for each combination according to:

ΣFIC = FIC_A_ + FIC_B_ = (C_A_/MIC_A_) + (C_B_/MIC_B_)

The drug interaction was scored as follows: synergy—FICI ≤ 0.5; no interaction—FICI > 0.5–4; antagonism—FICI > 4 [40].

### 4.5. Time–Kill Kinetics

Time–kill kinetics were established for compounds **2**, **5** and **10** in *E. faecium* ATCC 19434 and *E. faecalis* ATCC 29212. Prior to the experiments, the isolates were incubated on BHI agar supplemented with 5% calf blood at 37 °C overnight (ON). For each of the strains 1-2 colonies were transferred into 4 mL BHI broth and incubated ON at 37 °C with shaking (180 rpm). OD_600_ was measured, and then cultures were diluted with BHI broth to an OD_600_ of 0.05 and grown for approximately 2 h to reach the exponential phase. The exponentially growing cultures were diluted with BHI broth to OD_600_ 0.075, and 2.5 mL was transferred into 50 mL tubes and further diluted 2-fold with 2.5 mL BHI broth containing compound **2**, **5** or **10**, reaching a final concentration of peptidomimetic corresponding to 4× MIC for the respective strains (Table 2). 

One untreated control per strain was prepared. The inoculum was quantified by plating and subsequently enumerated. The initial inocula were approximately 10^7^ CFU/mL. Two controls were included, both without bacteria and one including the respective compound without bacteria. All tubes were incubated at 37 °C with shaking (180 rpm). Samples of 100 µL were taken after 1 h, 3 h and 5 h of exposure. Appropriate dilutions were spread onto BHI agar plates and incubated at 37 °C overnight, and then colonies were enumerated. Plates were incubated for 24 h and 48 h, and then checked for the appearance of slow-growing colonies. The experiments were performed with at least three biological replicates for each condition and time point.

### 4.6. Assessment of Membrane Integrity Using Flow Cytometry

Membrane integrity was assessed by using a propidium iodide (PI) uptake assay, as previously described [41]. In brief, 2 μL of stationary enterococcal cultures were transferred to Falcon round-bottom tubes (14 mL; Corning), each containing 2 mL fresh BHI medium. Cultures were grown to an OD_600_ of 0.2 at 37 °C with shaking (180 rpm). At OD_600_ of 0.2, compound **2** (at MBC), **5** (at MBC), **10** (at MBC) or nisin (25.6 μg/mL; 1× MIC) was added to the tubes and incubated for 5 min. Then, 15 μL of culture was transferred into 980 μL filtered phosphate-buffered saline, and then stained for 5 min with 5 μL of 0.1 mg/mL PI. After staining, the red fluorescence levels (FL3 channel) in cells were recorded by using a BD Biosciences Accuri C6 flow cytometer (Becton Dickinson). The settings of the flow cytometer were as follows: 25,000 recorded events at an FSC threshold of 15,000, and medium flow rate. The gating of the stained cell population and analysis of flow cytometry data were performed in CFlow (BD Accuri). The assessment of membrane integrity was performed in three biological replicates for each condition.

### 4.7. Statistics

The data were analyzed by using GraphPad Prism 8 (GraphPad Software Inc., San Diego, CA, USA) using one-way analysis of variance with a post hoc analysis of Dunnett’s multiple comparison tests. Log-transformation of the membrane integrity dataset was performed. For the statistical analysis, *p* < 0.05 was considered significant, and the degrees of statistical significance are presented as ★ *p* < 0.05, ★★ *p* < 0.01, and ★★★ *p* < 0.001.

### 4.8. Determination of Hemolytic Activity

A suspension of hRBCs (Single-donor human red blood cells—washed; catalogue No. IPLA-W3 from Innovative researchTM) was washed three times with PBS buffer and centrifuged for 5 min at 2500 rpm. A two-fold serial dilution of compounds in PBS buffer was prepared. Plates (conical-bottomed 96-well plates) with 3 replicate wells per dilution, containing 1% red blood cell suspension, were prepared and dosed with the test compound to achieve a final concentration of 400 μg/mL in a total volume of 100 μL. The plates were incubated (37 °C) for 60 min with mild agitation, and then the cells were pelleted by centrifugation at 1500 rpm for 5 min. Then, the supernatants (50 μL) were transferred to a fresh 96-well plate, and then the concentration of hemoglobin was detected by measuring the OD at 405 nm. The OD of cells incubated with 1% SDS achieved 100% hemolysis, while the OD of cells incubated with PBS buffer achieved 0% hemolysis. The concentration tested was 400 μg/mL (only average values are stated in Table 3). The percent hemolysis was calculated by using the formula:

Hemolysis(%) = 100 × [(A_sample_ − A_neg.control_)/(A_pos. control_ − A_neg.control_)]

### 4.9. Determination of Antiproliferative Activity on HepG2 Cell Line

The effect of peptidomimetics on mammalian cell viability was determined on the HepG2 cell line ATCC HB-8065. In brief, HepG2 cells were seeded into flat-bottom 96-well plates at a concentration of 5000 cells per well. The cells were incubated for 24 h in a humidified incubator (5% CO_2_, 37 °C). Subsequently, the medium was removed, and the cells were incubated for 48 h in a humidified incubator (5% CO_2_, 37 °C) with peptidomimetics in serial dilutions. An MTT assay was then performed; in brief, after incubation with the test compounds, the culture medium was removed and fresh medium with 0.2 mg/mL MTT was then added into each well. After incubation (3 h, 37 °C, 5% CO_2_), the medium with MTT was removed, and 200 μL dimethyl sulfoxide was added at once to each sample. The absorbance of MTT was measured using the spectrophotometer TECAN Infinite M1000 at 540 nm. The relative viability was calculated by using the formula:

OD_treated cells_ × 100/OD_control cells_

The IC_50_ values were calculated using the program Graph Pad Prism 5.0. For all compounds, the test range was 10–1280 μg/mL.

## Figures and Tables

**Table 2:** Minimum inhibitory concentrations for peptidomimetics and vancomycin against *E. faecium* and *E. faecalis* strains.

**No.**	***E. faecium***					***E. faecalis***		
	**D344R ^1^**	**ATCC** **19434**	**3978 ^1^**	**2961 ^1^**	**1798 ^1^**	**ATCC** **29212**	**38262 ^2^**	**39002 ^2^**
Subclass I								
**1**	4	8	4	2	2	32- > 32	16	32
**2**	2	2	2	2	2	2–4	4	2
Subclass II								
**3**	4–8	16–32	8	8	4–8	>32	32- > 32	32- > 32
**4**	4	8	4–8	4	4	>32	16	32
**5**	2	4	2	2	2	4–8	8–16	8–16
**6**	4	8	4	4	4	16	16	16
Subclass III								
**7**	4–8	8–16	4–8	2–4	2–4	8–16	>32	8–16
**8**	2	2–4	2	2	2–4	2–4	8–16	2
**9**	2	2–4	2–4	2–4	2–4	2–4	8	4
**10**	4	8	2–4	4	2–4	8–16	16	8
**11**	2–4	4	2–4	2–4	2–4	4	4–8	4–8
Vancomycin	2	1–2	16	>256	>256	2–4	1	1

*^1^E. faecium* D344R, 3978, 2961 and 1798: human clinical isolates; ^2^ *E. faecalis* 38262 and 39002: veterinary isolates.

**Table 3:** Correlation between hydrophobicity and effect on cellular viability.

No.	Luna C18(2) HST ^1^ (0–60% B; 10 min)	Hemolysis (at 400 µg/mL)	HepG2 IC_50_ (µg/mL)
Subclass I			
**1**	41.8	6.1%	9
**2**	51.6	98.4%	15
Subclass II			
**3**	40.2	0.6%	41
**4**	43.6	2.6%	42
**5**	46.6	9.4%	73
**6**	45.9	15.7%	42
Subclass III			
**7**	41.3	1.5%	50
**8**	45.9	28.8%	27
**9**	46.8	23.0%	15
**10**	47.2	6.9%	41
**11**	50.5	95.6%	22

^1^ Column used in analytical RP-HPLC gradient elution with a linear B concentration; the retention is converted into % MeCN.

## Supplementary Materials

The following are available online at https://www.mdpi.com/article/10.3390/ijms22115617/s1. Reference [42] is cited in the supplementary materials (denoted as reference [1] herein).
