# Supplementary material for: Peptide/β-Peptoid Hybrids with Activity against Vancomycin-Resistant Enterococci: Influence of Hydrophobicity and Structural Features on Antibacterial and Hemolytic Properties
Source: Int J Mol Sci. 2021 May 25;22(11):5617. doi: 10.3390/ijms22115617 (PMC8197855; doi:10.3390/ijms22115617)
Supplement: Supplementary file 1 [file ijms-22-05617-s001.zip › ijms-1184797-supplementary.pdf]

Supporting information

Peptide/β-peptoid hybrids with activity against vancomycin-resistant enterococci: Influence of hydrophobicity and structural features on antibacterial and hemolytic properties

Martin Vestergaard[1], Bolette Skive [1], Ilona Domraceva,[3] Hanne Ingmer[1] & Henrik Franzyk[2*]

1. Department of Veterinary and Animal Sciences, Faculty of Health and Medical Sciences, University of Copenhagen, Stigbøjlen 4, DK-1870 Frederiksberg C, Denmark.
2. Department of Drug Design and Pharmacology, Faculty of Health and Medical Sciences, University of Copenhagen, DK-2100 Copenhagen, Denmark.
3. Latvian Institute of Organic Synthesis, Aizkraukles 21, 1006, Riga, Latvia

Supplementary Table S1. Screening of 20 peptidomimetics against Enterococci.

Initial screening of peptidomimetics at a single concentration (32 µg/ml) against multiple *E. faecium* and *E. faecalis* strains. Compounds marked in blue were selected for further studies.

Inhibition at 48 h	Growth after 48 h	Growth after 24 h

		E. faecium				***E. faecalis***		
**No.**	**Sequence**	ATCC 19434	3978	2961	1798	ATCC 29212	37216	38262
**1**	Ac-[hArg-βNPhe]$_8$-NH$_2$							
	Ac-[hArg-βNspe]$_6$-NH$_2$							
**2**	Ac-[hArg-βNsce]$_6$-NH$_2$							
**3**	SpermineAc-[hArg-βNspe-Lys-βNspe]$_3$-NH$_2$							
	NDab-[hArg-βNspe-Lys-βNspe]$_3$-NH$_2$							
	TODA-[hArg-βNspe-Lys-βNspe]$_3$-NH$_2$							
**4**	H-[hArg-βNspe-Lys-βNspe]$_4$-NH$_2$							
	Ac-[hArg-Nspe-Lys-Nspe]$_3$-NH$_2$							
**5**	Ac-[hArg-βNsce-Lys-βNspe]$_3$-NH$_2$							
**6**	Cinn-[hArg-βNspe-Lys-βNspe]$_3$-NH$_2$							
	H-(NLys-Phe)$_8$-NH$_2$							
	SpermineAc-(Lys-βNPhe)$_6$							
	Ac-(Lys-βNspe)$_8$-NH$_2$							
**7**	H-[Lys-βNPhe(F)]$_8$-NH$_2$							
**8**	H-[Lys-βNPhe(F$_3$)]$_6$-NH$_2$							
**9**	H-[Lys-βNCha]$_8$-NH$_2$							
**10**	Oct-[Lys-βNspe]$_6$-NH$_2$							
**11**	Lau-[Lys-βNPhe]$_6$-NH$_2$							
	Ac-[NLys-Trp]$_8$-NH$_2$							
	Ac-[βNLys-Trp]$_8$-NH$_2$							

Abbreviations: Ac = acetyl; Cinn = cinnamoyl; hArg=homoarginine; Lau = lauroyl; βNCha = N-cyclohexylmethyl-β-alanine; NLys = N-(4-aminobutyl)-glycine; βNLys = N-(4-aminobutyl)-β-alanine; βNPhe = N-benzyl-β-alanine; βNPhe(F) = N-(4-fluorobenzyl)-β-alanine; βNPhe(F3) = N-(3,4,5,-trifluorobenzyl)-β-alanine; βNsce=N-(S)-1-cyclohexylethyl-β-alanine; βNspe=N-(S)-1-phenylethyl-β-alanine; Oct = octanoyl; SpermineAc = $H_2N(CH_2)_3NH(CH_2)_4NH(CH_2)_3NHCH_2(C{=}O)$-; TODA = $H_3CO(CH_2)_2O(CH_2)_2OCH_2(C{=}O)$-.

Supplementary Table S2. Drug combinations of peptidomimetics with conventional antibiotics.
N.D.: Not determined

Antibiotic	Peptidomimetic (+/- 0.5x MIC)	MIC µg/ml			
		E. faecium ATCC 19434	*E. faecalis* ATCC 29212	*E. faecium* 2961	*E. faecium* 1798
Vancomycin	-	1-2	2-4	>256	>256
	+ **2**	2	4	>256	>256
	+ **5**	2	4	>256	>256
	+ **10**	2	2	>256	>256
Gentamicin	-	64	16	N.D.	N.D.
	+ **2**	64	16	N.D.	N.D.
	+ **5**	32	16	N.D.	N.D.
	+ **10**	64	16	N.D.	N.D.
Ciprofloxacin	-	4-8	0.5-1	N.D.	N.D.
	+ **2**	4-8	1	N.D.	N.D.
	+ **5**	4-8	0.5-1	N.D.	N.D.
	+ **10**	4-8	0.5-1	N.D.	N.D.
Linezolid	-	2	2	N.D.	N.D.
	+ **2**	2	2	N.D.	N.D.
	+ **5**	2	2	N.D.	N.D.
	+ **10**	2	2	N.D.	N.D.
Rifampicin	-	8	1	N.D.	N.D.
	+ **2**	8	1	N.D.	N.D.
	+ **5**	8	1	N.D.	N.D.
	+ **10**	1-2	0.5-1	N.D.	N.D.
Azithromycin	-	4-8	1-2	N.D.	N.D.
	+ **2**	4-8	1	N.D.	N.D.
	+ **5**	4-8	1-2	N.D.	N.D.
	+ **10**	4-8	1-2	N.D.	N.D.

Supplementary Table S3. Minimum bactericidal concentration for three selected peptidomimetics.

Compound	MV388 (µg/ml)	MV269 (µg/ml)
**2**	4-8	8
**5**	128	16
**10**	16	32

Supplementary Table S4. Strains used in this study.

Identity no.	Strain name	Source
MV270	*E. faecium* D344R	[1]
MV388	*E. faecium* ATCC 19434	ATCC
MV389	*E. faecium* 3978	Human clinical isolate
MV390	*E. faecium* 2961	Human clinical isolate
MV391	*E. faecium* 1798	Human clinical isolate
MV269	*E. faecalis* ATCC 29212	ATCC
MV392	*E. faecalis* 37216	Veterinary isolate
MV393	*E. faecalis* 38262	Veterinary isolate
MV394	*E. faecalis* 39002	Veterinary isolate

Reference:

1. Williamson, R.; Le Bouguénec, C.; Gutmann, L.; Horaud, T. One or two low affinity penicillin-binding proteins may be responsible for the range of susceptibility of *Enterococcus faecium* to benzylpenicillin. *Microbiology* **1985**, *131*, 1933-1940, doi:10.1099/00221287-131-8-1933.
